# A Case Report of Candida-Induced Emphysematous Gastritis

**DOI:** 10.7759/cureus.47870

**Published:** 2023-10-28

**Authors:** Natalia C Pinto, Jorge Nadal Bosch, Yilen K Ng-Wong, Michael Menowsky, Ryan Shine, Javier Malcom, Mario Moya, Julian Galindo, Samuel Serna

**Affiliations:** 1 Internal Medicine, University of Texas Rio Grande Valley, Edinburg, USA; 2 Emergency Medicine/Critical Care, Doctors Hospital at Renaissance/University of Texas Rio Grande Valley, Edinburg, USA; 3 Surgery, University of Texas Rio Grande Valley, Edinburg, USA; 4 Medical Information, Doctors Hospital at Renaissance, Edinburg, USA; 5 Radiology, Doctors Hospital at Renaissance, Edinburg, USA; 6 Internal Medicine, Universidad Libre de Cali, Cali, COL

**Keywords:** antibiotics, ischemic colitis, clostridium difficile, ischemic bowel, diabetic ketoacidosis, candida spp, emphysematous gastritis

## Abstract

Emphysematous gastritis is a rare entity that has not much literature available. It is known to manifest as a diffused wall inflammation and air within the wall of the stomach and has been associated with gas-forming organisms.

We present a complex case of a middle-aged woman with a previous history of fulminant *Clostridium difficile* complicated with colectomy and diverting colostomy. She was admitted due to diabetic ketoacidosis, later complicated with worsening abdominal pain, and a CT scan of the abdomen and pelvis without contrast revealed findings consistent with ischemic bowel, severe pneumatosis intestinalis, and extensive portal venous gas. A stomach biopsy revealed hemorrhagic necrosis; a Gomori methenamine silver stain was compatible with fungal organisms, *Candida species*, correlating with Candida emphysematous gastritis. This case highlights the importance of early diagnosis of this syndrome in order to provide appropriate management, and early identification, to improve survival.

## Introduction

Emphysematous gastritis is a rare medical condition initially described in 1889, characterized by the presence of air within the stomach wall and diffuse wall inflammation attributed to gas-forming microorganisms [1,2]. While there are only a few documented cases in the English literature, even fewer are specifically associated with Candida species. Currently, there are no established guidelines for the management of emphysematous gastritis. The most commonly implicated pathogens include Escherichia coli, Enterobacter species, Pseudomonas aeruginosa, Clostridium perfringens, and Staphylococcus aureus. However, in exceptionally rare instances, Candida species have been reported as the causative agent [3]. Mortality rates associated with emphysematous gastritis are substantial, with a reported rate of 60%, which increases in cases requiring surgical intervention [4]. We present a challenging medical case involving a middle-aged female patient with a prior medical history notable for fulminant Clostridium difficile infection (CDI) that necessitated colectomy and diverting colostomy. This patient was admitted to our medical facility exhibiting non-specific symptoms, notably the persistence and exacerbation of abdominal pain. Subsequent evaluation led to the diagnosis of emphysematous gastritis induced by Candida. 

## Case presentation

The patient is a 41-year-old female with a medical history notable for type 2 diabetes mellitus, obesity, and a previous occurrence of fulminant Clostridium difficile colitis a decade prior to the current presentation. Notably, the diagnosis of the antecedent fulminant CDI was based on the fulfillment of established criteria, including the presence of severe diarrhea, leukocytosis, and elevated creatinine levels. Diagnostic confirmation was further substantiated through the utilization of imaging studies and colonoscopy, revealing characteristic findings such as colonic wall thickening and pseudomembranes. Given the rapid progression of symptoms and the severe nature of the infection, emergent surgical intervention in the form of a colectomy and diverting colostomy was undertaken. In the present hospitalization, the patient presented to the emergency department with a two-day history of generalized weakness, abdominal pain, nausea, vomiting, and diarrhea. The abdominal pain was described as a persistent burning sensation located in the epigastrium, with no specific exacerbating or alleviating factors. The patient reported 2-3 bilious emetic episodes without hematemesis and diarrhea characterized by 4-5 episodes of brown loose stool, with no evidence of melena or hematochezia. There were no other associated symptoms reported by the patient in the emergency room.

During physical examination upon admission, vital signs did not reveal any warning signs, with a temperature of 98.2, blood pressure of 109/67 mmHg with a mean arterial pressure of 81 mmHg, pulse of 98 beats per minute, 10 respirations per minute, and oxygen saturation of 99% on room air. The patient was alert and oriented to person, time, and place, and cardiovascular and pulmonary examination was unremarkable. Abdominal exam revealed an obese abdomen, soft, mildly tender to superficial palpation in the epigastrium, normal bowel sounds, no guarding or rebound tenderness. Initial laboratory data revealed leukocytosis, hyperglycemia, electrolyte derangements, anion gap metabolic acidosis, and ketones in the blood (Tables 1, 2). Complete urinalysis showed glucose, ketones, blood, protein, nitrate, leukocyte esterase, WBCs, RBCs, and many bacteria, initial urine culture showed ESBL Klebsiella pneumoniae, with negative blood cultures. The initial CT scan of the abdomen and pelvis without contrast showed mild distention of the partially imaged distal esophagus with an air-fluid level, heterogeneous fat, and soft tissue attenuation mass like an abnormality in the anterior right lower quadrant which was indeterminate, post-operative changes from colectomy with right paracentral anterior pelvic ileostomy, parastomal herniation of small bowel loops and several adhesions to the walls of the hernia sac, with a non-obstructive small bowel gas pattern.

**Table 1:** Laboratories taken upon admission

Laboratories	Results	Normal Values
Sodium	131 mEq/L	135-145 mEq/L
Potassium	5.5 mEq/L	3.5-4.5 mEq/L
Bocarbonate	10 mmol/L	21-31 mmol/L
Chloride	101 mEq/L	95-105 mEq/L
Creatinine	4.5 mg/dL	0.6-1.2 mg/dl
Blood urea nitrogen (BUN)	87 mg/dL	5-20 mg/dl
AST	10 IU/L	12-37 U/L
ALT	12 IU/L	15-65 U/L
ALK	92 IU/L	50-136 U/L
Albumin	3 g/dl	3.7-4.9 g/dL
Bilirubin	0.2 mg/dl	1.2 mg/dl
Lactic acid	2.70 mmol/L	0.5- 1.9 mmol/l
Blood glucose	410 mg/dl	70-110 mg/dl
WBC	12.7 th/ul	4.8-10.9 th/dl
Troponin	0.04 ng/ml	0.00-0.04 ng/ml
Serum ketones	Large	Negative

**Table 2:** Arterial blood gas upon admission

abg	Results	Normal values	
pH	7.31	7.35-7.45	
CO_2_	34 mmHg	35-48 mmHg	
O_2_	94 mmHg	83-108 mmHg	
HCO_3_	17 mol/l	18-23 mmol/l	

The patient was initiated on a treatment regimen comprising fluid resuscitation, broad-spectrum antibiotics, intravenous insulin infusion, and electrolyte replacement. This approach led to the gradual improvement of serum glucose levels and the normalization of lactic acid levels, subsequently allowing the transition to subcutaneous insulin therapy. The patient's condition stabilized, and she was transferred to a telemetry unit.

**Figure 1:**
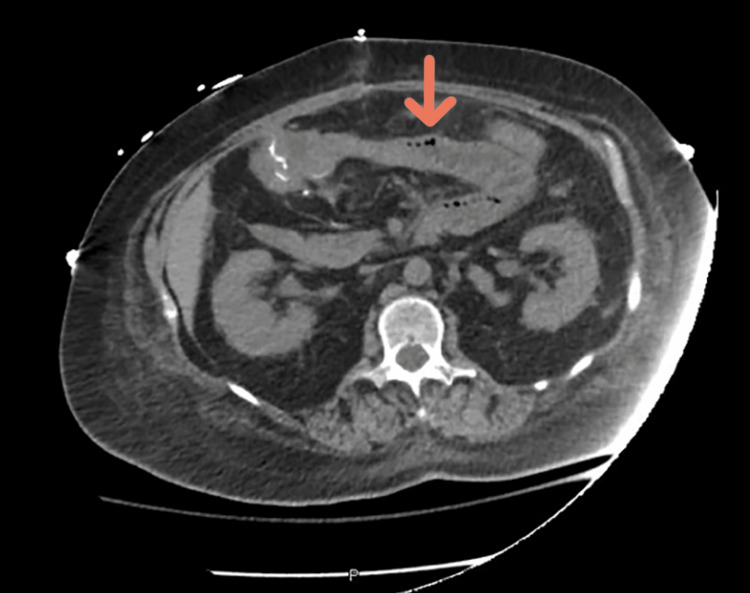
CT scan of the abdomen showing Pneumatosis intestinalis

**Figure 2:**
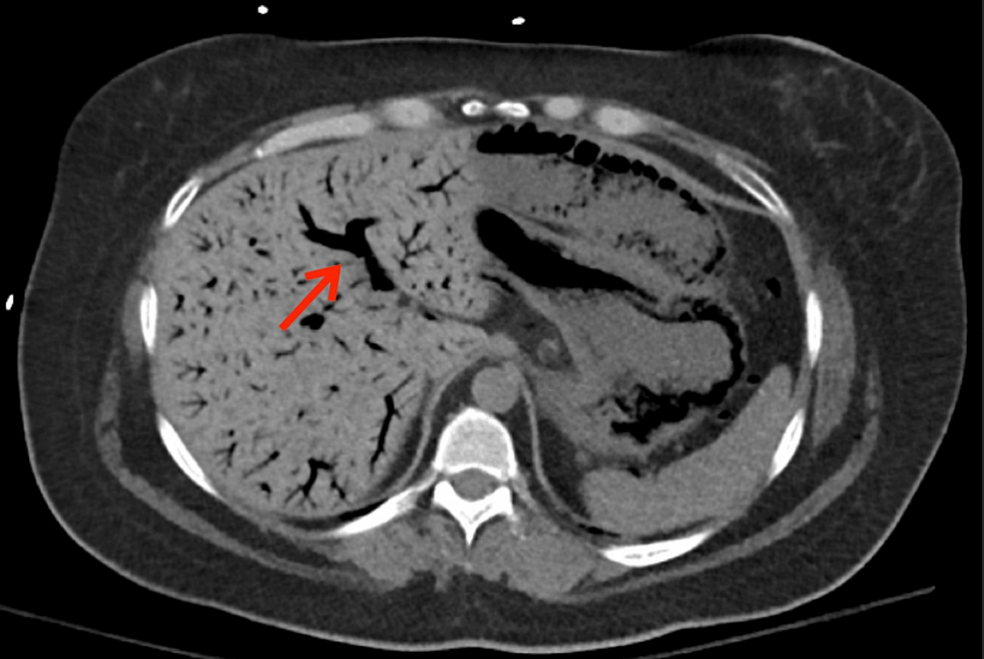
CT Scan of the abdomen showing portal venous gas

**Figure 3:**
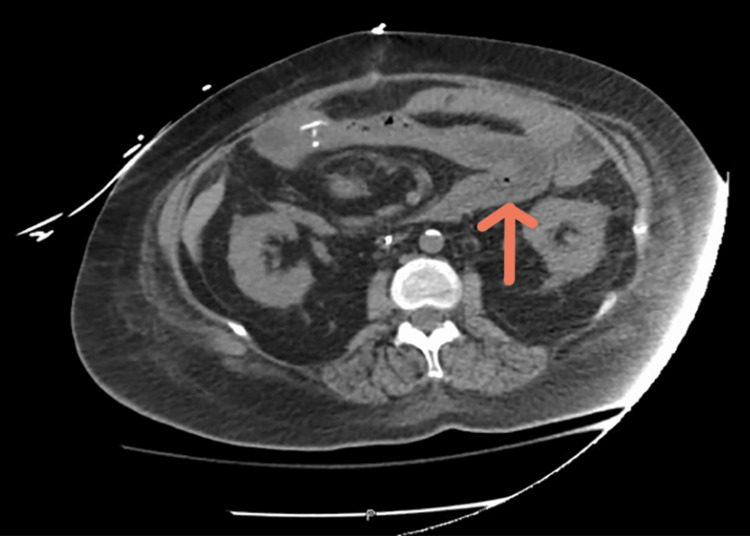
CT scan of the abdomen showing widespread ischemic bowel

However, on the fourth day of hospitalization, the patient developed severe abdominal pain. Clinical examination revealed tenderness in all quadrants, guarding, rebound tenderness, and abdominal distension, suggestive of acute abdomen. The patient underwent a second CT scan of the abdomen and pelvis without contrast, revealing evidence of widespread ischemic bowel with severe pneumatosis intestinalis and extensive portal venous gas in the liver which was new compared to the prior CT scan (Figures 1-3).

**Figure 4:**
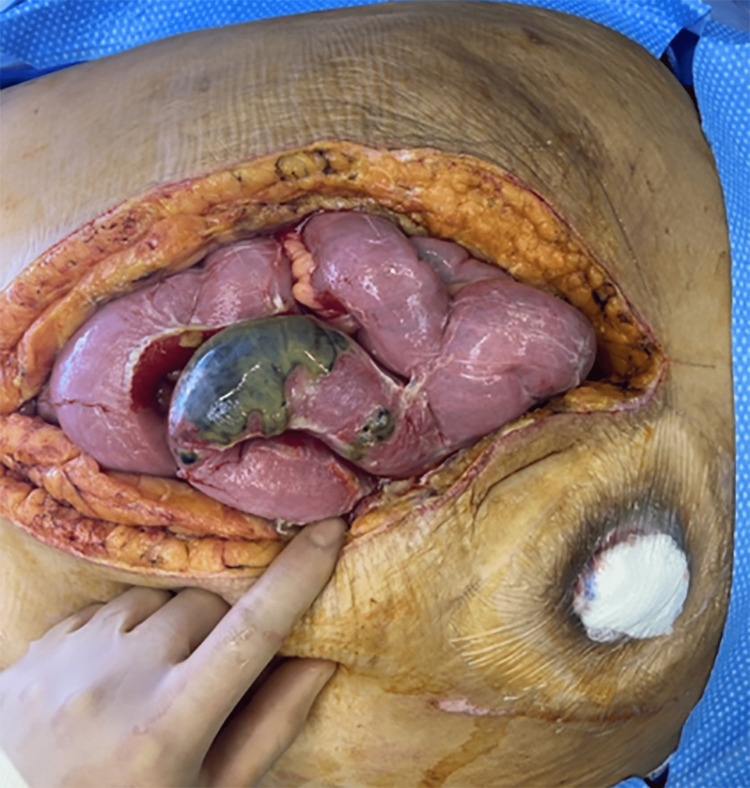
Emergent exploratory laparotomy showing bowel necrosis

Immediate consultation with the general surgery team was sought, and the patient was taken for an urgent exploratory laparotomy, which involved enterolysis, excision of left lower quadrant soft tissue, and the placement of an ABTHERA (Ò) wound vacuum (Figure 4). Subsequently, the patient was transferred to the intensive care unit (ICU). 

The patient's clinical course presented formidable challenges. On the sixth day of her hospitalization, her condition deteriorated significantly, ultimately leading to septic shock, aggravated acidosis, and a pronounced clinical decline. In response, the medical team initiated aggressive measures, including fluid resuscitation and vasopressor support. Recognizing the severity of the situation, a second urgent exploratory laparotomy was performed, revealing extensive bowel necrosis, necessitating a segment of the small bowel to be resected and left in discontinuity.

In light of these critical developments and the presence of worrisome necrotic findings within the stomach wall, the expertise of the gastrointestinal team was enlisted. Esophagogastroduodenoscopy (EGD) was carried out to obtain a more detailed visual assessment. The EGD uncovered substantial mucosal necrosis of the small bowel. The potential presence of Mucormycosis was also taken into consideration. To establish a definitive diagnosis, a biopsy of stomach tissue was performed.

Pending histopathology results, the patient was empirically initiated on amphotericin B. However, the results from the small bowel tissue biopsy exhibited diffuse hemorrhagic necrosis and a special stain demonstrated fungal organisms consistent with Candida species. Importantly, the biopsy findings excluded Mucormycosis, Helicobacter pylori infection, or malignancy. Consequently, the patient's treatment was adjusted to micafungin therapy, given that the organism identified on biopsy belonged to the Candida species.

On the eighth day of hospitalization, the patient underwent a repeat abdominal exploration, which included an abdominal washout, small bowel resection left in discontinuity, and temporary abdominal closure. Subsequently, on the eleventh day, the patient underwent another abdominal re-exploration, during which re-anastomosis was performed, followed by abdominal closure. By the twelfth day, the patient exhibited sufficient clinical improvement to enable successful weaning off vasopressors and sedation, ultimately culminating in successful extubation.

Due to this patient’s clinical presentation, and prior history of fulminant C. difficile associated with an immunosuppressed state like uncontrolled diabetes mellitus, which already provides a favorable environment for colonizing species as Candida spp, associated with the CT scan findings, histopathology from small bowel tissue biopsy, and positive rapid response to echinocandins, we could infer that the most likely diagnosis was candida induced emphysematous gastritis, a rare condition that is often fatal. 

## Discussion

In the context of intramural gas within the stomach, it is essential to differentiate between gastric emphysema and emphysematous gastritis. Gastric emphysema, a typically benign condition, arises from disruptions in the gastric mucosa, permitting the entry of air into the stomach wall. Causative factors may include severe vomiting, instrumentation or endoscopy, gastric ischemia, or the dissection of air from the mediastinum. Notably, patients with gastric emphysema do not typically present with acute abdominal symptoms, and the approach to management is generally conservative and supportive, resulting in an excellent prognosis.

Conversely, emphysematous gastritis represents an infectious process driven by gas-forming microorganisms, which can encompass a range of pathogens, such as Streptococcus species, Escherichia coli, Enterobacter species, Clostridium species, Pseudomonas aeruginosa, Staphylococcus aureus, Candida species, and Mucor species. Patients afflicted with emphysematous gastritis often exhibit severe abdominal pain, nausea, vomiting, hematemesis, and systemic manifestations like fever [5,6].

In the case we present, a patient with underlying risk factors compromising the stomach's natural infection barriers, including diabetes and a history of recurrent Clostridium difficile colitis, status post abdominal colectomy with ileostomy, was admitted due to diabetic ketoacidosis (DKA) accompanied by abdominal pain. The initial abdominal CT scan did not reveal any abnormalities; however, owing to the worsening abdominal pain, a repeat CT of the abdomen was conducted, unveiling compelling evidence of widespread ischemic bowel, pneumatosis intestinalis, and extensive portal venous gas, signifying a complex clinical scenario. Surgical intervention was deemed necessary, and the patient was ultimately diagnosed with Candida-induced emphysematous gastritis, emphasizing the gravity of this condition and the critical importance of timely and comprehensive diagnostic evaluation.

## Conclusions

We present a complex case of Candida spp emphysematous gastritis in a patient with multiple comorbidities, including a previous episode of DKA and an intra-abdominal infection necessitating surgical intervention. Given the absence of established guidelines for the management of this rare condition, it underscores the significance of this case as a potential avenue for research. Such circumstances increase the risk of a typically benign colonizing microorganism to manifest in an aggressive and potentially fatal manner. Early diagnosis of this condition becomes paramount, as it can facilitate timely and appropriate management, whether through conservative or surgical approaches, with the potential to enhance patient survival. This case highlights the need for further investigation in the field.
